# Hutchinson-Gilford Progeria Syndrome: A Literature Review

**DOI:** 10.7759/cureus.28629

**Published:** 2022-08-31

**Authors:** Aselah Lamis, Shiza W Siddiqui, Tejaswini Ashok, Nassar Patni, Mahejabeen Fatima, Asiff Nathi Aneef

**Affiliations:** 1 Research, Dubai Medical College, Dubai, ARE; 2 Internal Medicine, Jagadguru Sri Shivarathreeshwara (J.S.S) Medical College, Mysore, IND; 3 Internal Medicine, Deccan College of Medical Sciences, Hyderabad, IND; 4 Trauma and Emergency, All India Institute of Medical Sciences, Patna, Patna, IND

**Keywords:** clinical trial, lonafarnib, bone mineralization, generalized osteopenia, atherosclerosis, lmna mutation

## Abstract

Hutchinson-Gilford progeria syndrome (HGPS) is a premature aging condition that involves genetic mutations, resulting in debilitating phenotypic features. The present state of knowledge on the molecular pathways that contribute to the pathophysiology of HGPS and the techniques being tested *in vitro* and *in vivo* to combat progerin toxicity have been discussed here. Nuclear morphological abnormalities, dysregulated gene expression, DNA repair deficiencies, telomere shortening, and genomic instability are all caused by progerin accumulation, all of which impair cellular proliferative capability. In addition, HGPS cells and preclinical animal models have revealed new information about the disease's molecular and cellular pathways and putative mechanisms involved in normal aging. This article has discussed the understanding of the molecular pathways by which progerin expression leads to HGPS and how the advanced therapy options for HGPS patients can help us understand and treat the condition.

## Introduction and background

Hutchinson-Gilford progeria syndrome (HGPS) is a segmental "premature aging" condition in which children show phenotypes that may reveal information about the aging process at both the cellular and organismal levels [1]. According to Progeria Research Foundations, relatively 350-400 children are estimated to be living with progeria worldwide, irrespective of sex and race [2]. It affects 1 in 20 million people approximately [2]. Jonathan Hutchinson discovered it in 1886, but the disease was poorly understood [3]. In 2003, a group of French researchers discovered point mutations in the LMNA genes [3]. Progeria, a laminopathy, was caused by the mutation of lamin A which, encoded by the LMNA gene, supports the protein complexes that help keep the cell nucleus stable and the genomes intact [4,5]. However, when Lamin A undergoes mutation, it causes destabilization of the nucleus and DNA damage, ultimately leading to the aging effects [4, 5]. Children with progeria have a disproportionately small face compared to the head, abnormally prominent eyes, a narrow nasal bridge and tip, a small jaw, malformation and crowding of the teeth, and micrognathia [6]. Loss of subcutaneous fat delayed the eruption and loss of primary teeth, irregular skin with small outpouching over the abdomen and upper thighs, alopecia, nail dystrophy, coxa valga, and progressive joint contractures are all common symptoms [6]. Low-frequency conductive hearing loss, dental crowding, and a partial absence of secondary tooth eruption were discovered later [6]. It is standard for a child's motor and mental development to be normal [6]. Between the ages of six and 20 years, death occurs as a result of complications of serious atherosclerosis, either cardiac disease (myocardial infarction or heart failure) or cerebrovascular disease (stroke) [6]. Typical findings include generalized osteopenia, resorption of bone seen around the phalanges and distal clavicle, fish-mouth vertebral bodies, and wide metaphyses with narrowed diaphyses, including coxa valga and hip dysplasia [7]. A range of craniofacial structures, such as abnormal mandibular condyles, hypoplastic articular eminences, optic nerve kinking, and soft tissue abnormalities, have been identified on CT and MRI [7]. In histological findings, the skin from sclerotic and firm areas shows the characteristics of scleroderma in the early stages with acanthosis of the epidermis. Thickened collagen bundles can be seen in the dermis extending to the subcutaneous tissue [7]. Mild perivascular infiltrate may be present, and the amount of mucopolysaccharides acid is increased [7]. As the disease progresses, there is a marked reduction in subcutaneous fat. Blood vessels appear to be thickened, leading to narrowed vascular lumen [7]. Lonafarnib is a farnesyltransferase inhibitor that prevents the buildup of defective progerin or progerin-like protein in the body [8]. Lonafarnib was approved for the first time in the United States in November 2020 to reduce the risk of mortality in HGPS and to treat processing-deficient progeroid laminopathies [8]. From the above statement, it can be understood that HGPS can prove to be a fatal condition in the long run and can confer poor quality of life to the patients. This review article aims to highlight the genetic background of HGPS and explore the treatment for the same.

## Review

Pathophysiological mechanism underlying the development of HGPS

HGPS is a sporadic, autosomal dominant disorder caused almost entirely by de novo point mutations of the LMNA gene in codon 608 of exon 11 on chromosome 1 [8, 9]. The LMNA gene enciphers three components of the nuclear laminae, which are proteins called lamin A (LA), lamin C (LC), and lamin 10, a complex molecular interface within the inner nuclear membrane [10]. The lamina has now proven to play a significant role in cell division, chromatin organization, DNA replication, nuclear shape, and transcription [11]. The LMNA mutation in its typical form in HGPS is a C-T nucleotide substitution, creating a cryptic splice donor site in position 1824, causing no difference in encoded amino acids. When activated, this site leads to mRNA lacking 150 nucleotides [12]. This mRNA is then translated into "progerin," a deviant protein with a 50-aminoacids internal deletion near the C terminus [12]. Most differentiated cells express LA, which affects the integrity of both the nuclear structure and function [12].

**Figure 1:**
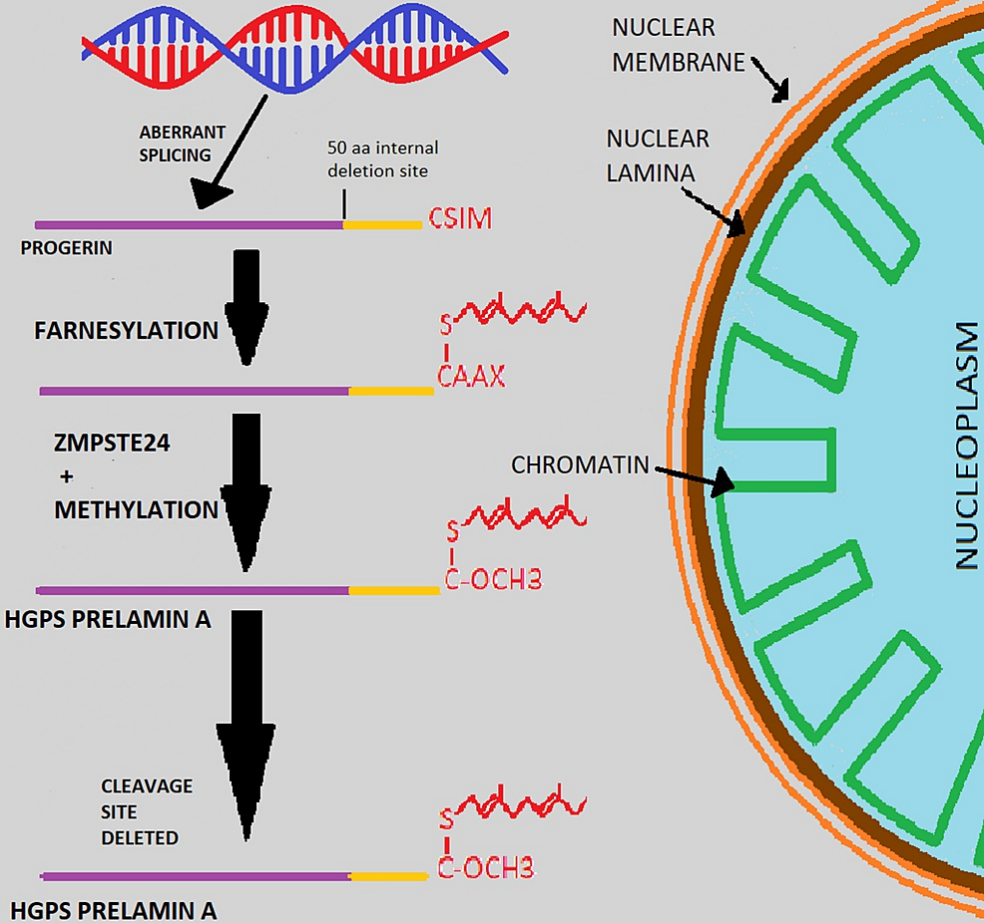
The pathophysiological mechanism of HGPS. HGPS: Hutchinson-Gilford progeria syndrome; aa: Amino acids. Image Credits: Aselah Lamis

Progerin presumably has a dominant-negative effect on the nuclear function of the LA-expressing cells. It is also hypothesized to harm other critical processes such as cell division, DNA replication, and gene transcription [13-15]. CAAX motif is defined as a cysteine molecule accompanied by two aliphatic amino acids and a final "X" remnant [15]. Generally, prelamin A consists of the CAAX tetrapeptide motif at the C-terminus [16]. The tetrapeptide, with the help of the enzyme farnesyltransferase (FTase), signals a specific inclusion of a 15-carbon farnesyl isoprenoid lipid group to the cysteine [16]. The amino acids of the CAAX define the specific adding of an isoprenyl group with alanine, glutamine, methionine or serine with the help of FTase signaling modification or with leucine, with the help of enzyme geranylgeranyltransferase (GGTase) will signal the addition of 20-carbon geranylgeranyl isoprenoid group [17]. The CAAX motif for LA is CSIM [16, 17]. Prelamin A interaction with the nuclear membrane is promoted by farnesylation and subsequent CAAX signaling modification [18]. Following farnesylation, the three-terminal amino acids (AAX) are removed, and the cysteine group at the c-terminal undergoes methyl esterification [19, 20]. Uniquely, a second cleavage occurs within the nucleus that eliminates added 15 C - terminal amino acids, including the farnesylated cysteine from the mature protein [16]. Presumably, the prelamin A is released from the nuclear membrane and incorporated into the nuclear lamina after this end cleavage stage and the loss of the farnesyl anchor [16]. Even though preprogerin can be farnesylated in HGPS, the internal deletion of amino acids 606-656 eliminates the endoprotease recognition section required for the final cleavage phase [16]. The case that transmutations in ZMPSTE24 generate an extreme shape of mandibuloacral dysostosis, one of many laminopathies with phenotypic similarities to HGPS, demonstrates the relevance of this cleavage [20]. The human analog of yeast STE24, ZMPSTE24, is important for the end cleavage of Lamin A [20]. Progerin maintains its farnesyl group since the last cleavage cannot occur in LMNA G608G, stabilizing progerin interactions with the inner nuclear membrane [21-23]. Progerin will cause the nuclear envelope (NE) to expand inward in this case, increasing the surface area available to accommodate the excess progerin accumulation in HGPS (Figure 1) [21-23].

Clinical manifestations caused by HGPS

Typically, the replicative capacity in an accelerated aging syndrome is reduced [24-26]. Allsopp RC et al. desired a diagnosis of whether telomeres are likely to be shortened in such diseases [27]. In order to test this, they obtained lengths of telomere restriction fragment (TRF) within fibroblast cultures from patients suffering from HGPS. They analyzed a comparison with healthy individuals of the same age [26]. They established that all five cell strains of pooled mean TRF length obtained from those progeria donors had decreased propagative capacity ex vivo compared to the corresponding values in the five young normal donors (p <0.001) [26]. Telomere length and replicative ability were both reduced in fibroblasts from progeria donors, indicating that the biomarker of cellular aging is telomere length [26]. In fibroblasts obtained from progeria, the patients' parents showed no signs of premature aging, and the mean TRF duration was comparable to that of age-matched average donors. This supports the idea that Hutchinson-Gilford progeria is produced by a de novo autosomal dominant mutation [26].

The irregular telomere length regulation in a parental germ-line clone, an elevated cell turnover rate during progeric individual's growth, or an abnormally elevated rate of telomere deficit with every cell division could all contribute to progeric fibroblast's short telomeres [26]. They compared telomere deficit during fibroblast aging from three patients suffering from progeria ex vivo to that of normal donors of the same age to assess the latter possibility [26]. In progeria patients' fibroblasts, there was a slight but analytically inconsequential rise in the incidence of telomere deficit, although other factors also likely play a role in the shortened telomeres observed in these cells [26]. Patients with the typical HGPS gene transmutation appear normal at birth, and by the age of one year, they develop remarkably similar signs and symptoms [27,28]. Alopecia, severe loss of subcutaneous fat, lack of weight gain, skeletal manifestations that include contractures, coxa valga, osteolysis, shortened clavicles, and extreme short stature are all characteristics of this phenotype [27, 28]. Mineralization has been identified in an uneven pattern, particularly at the metaphysis of the long bones [27].

Rigidity is a structural property that determines a bone's ability to withstand axial stresses, bending, and torsional moments [29]. It is the fundamental outcome of bone tissue modulus (a role of bone mineral density) and the cross-sectional framework of bone. It has been found to be significantly irregular in HGPS patients compared to healthy controls [29]. A study published in 2011 performed various techniques, such as Stress-Strain Index (SSI) by peripheral quantitative CT (pQCT), a CT-based method, as an indicator of bone-building strength, to provide assessments of trabecular and cortical bone, and to calculate the cross-sectional (axial, bending and torsional) rigidities at the radius, respectively [29]. It was found that the axial rigidity was 40% lesser at the diaphysis and metaphysis of the radius, the bending and torsional rigidities were 66% lesser, and SSI was notably lower in HGPS children when compared to normal controls proving that HGPS tends to mainly influence the structural geometry which is suggestive of skeletal dysplasia [29]. Further evidence proving skeletal abnormality in HGPS children includes the aberrant arrangement of decreased mineralization of the long bones [28, 29]. In senile osteoporosis, the primary pathophysiology includes a marked increase in bone resorption leading to an initial fall in bone density and significant bone formation reduction in the elderly, while in HGPS patients, the bone formation was in the normal range [30]. The bone resorption markers were also in the typical range [30]. As a result, traditional biomarkers for senile osteoporosis, a multi-factorial condition, do not apply in HGPS, where the disease is caused by the downstream effects of a single protein variant, progerin [30]. A study that was published in 2010 on the correlation between progeria and atherosclerosis between HGPS and typical adult cardiovascular disease (CVD) found that in the HGPS patient samples, a spectrum of early-to-late stage plaques was found similar to geriatric CVD [30]. Inflammation, calcification, and plaque erosion and/or rupture were exhibited on the arterial lesions of both typical atherosclerosis and HGPS patients [31]. Smaller atheromatous cores of the HGPS lesions can be caused due to the lack of dyslipidemia and hypercholesterolemia, even though they tend to appear relative to typical atherosclerosis [31]. It is likely that in HGPS vascular pathology, multiple cell types are involved, and the macrophages, as well as the VSMC, despite their limited capacity for cell renewal, may play a role as well [31].

Progerin accumulation can contribute to the development of thickening of the adventitia in small, medium, and large arteries, ultimately resulting in decreased intimal plaque formation potential, decreased vascular compliance, and increased vessel stiffness [31]. As adventitial fibrosis keeps proceeding, the stiffness of the aorta will cause an increase in the afterload of the cardiac muscles leading to left ventricular hypertrophy [31]. Adventitial fibrosis, which can also be caused by chronic ischemia and luminal narrowing, may occur as a result of modifications in collagen deposition and regrouping in response to inflammation or mechanical stress [32]. Ex vivo, HGPS fibroblasts have lower feasibility, are more vulnerable to oxidative damage, and the nuclear lamina's capacity to rearrange under mechanical stress is significantly reduced [33-35]. Olive M et al. proved that progerin accumulation within the vasculature implies a direct and indirect influence on progressive cardiovascular disease by identifying its presence in the intimal plaques and arterial walls of the aorta, coronary arteries, arterioles, and veins of HGPS patients [31]. There was a dramatic accumulation of progerin identified in the VSMC, and adventitia concentrated into a thick band-like structure at the site of the NE [31].

By illustrating progerin exists in the coronary arteries of healthy aging individuals and rises with age, we also recognize a new factor in the traditional aging process [36-38]. Inhabiting vascular cells in vivo use the cryptic splice site in exon 11 of LMNA infrequently [31]. In normal fibroblast lines, Progerin-positive cells manifest limitations in the mitosis that escalate with passage number [31]. This finding supports the theory that progerin-induced mitotic defects are linked to normal aging [31]. The adventitia had the maximum number of progerin-positive cells in non-HGPS arteries, suggesting that some vessel abuse can begin in this deep layer of the vessel and then cause distress to the intima, indicating plaque formation [31].

Management options for HGPS

To correct the defects in HGPS, a variety of treatment strategies have been proposed, including: (i) directly "repairing" the transmutation; (ii) inhibiting pre-mRNA mutant splicing generating progerin mRNA formation; (iii) reducing the toxic levels of isoprenylated and methylated progerin; (iv) to generate progerin removal; and (v) to reduce the harmful overdue buildup of progerin [39].

Reduction of Progerin Downstream Toxic Effects

Nuclear shape malformation, reactive oxygen species (ROS) production, aggregation of oxidized proteins, mitochondrial impairment, cell senescence, and NF-kB stimulation, resulting in elevating levels of secretion of the proinflammatory cytokines IL-6, CXCL1, and TNF-α, are just a few of the altered downstream pathways caused by progerin accumulation that have been described [40]. In progeroid fibroblasts, the ROS scrounger N-acetyl cysteine (NAC) lessened the number of unrepairable DNA double-strand breaks (DSB) and boosted their expansion in culture [40]. Rho-associated protein kinase (ROCK) has also been found to regulate mitochondrial ROS formation via altering the connection between cytochrome c and Rac1b [41]. Ex vivo management of HGPS fibroblasts with the ROCK inhibitor (Y-27632) reduced ROS levels. In addition, it caused mitochondrial function reclamation, as well as a decrease in the recurrence of aberrant nuclear morphology and DSB [41]. Reactivating NRF2, whose transcriptional activity is hindered in HGPS cells, reduced the elevated levels of ROS and oxidative stress, resulting in improvements in cellular HGPS abnormalities [42]. MG132, interestingly, appears to be a potentially beneficial medication for preventing oxidative stress in HGPS cells via activating the NRF2 signaling pathway [43]. However, mitochondrial malformation has been observed in both HGPS fibroblasts and HGPS mice models [44, 45]. To correct the mitochondrial deficiency, Xiong ZM et al. found that treating HGPS cells with an antioxidant called methylene blue (known for its stimulation of mitochondrial function) enhances the mitochondrial physiology along with premature aging phenotypes in HGPS cells, such as misregulated gene expression, nuclear morphology, and perinuclear heterochromatin loss [45]. In LmnaG609G/+ mice, mitochondrial malformation causes increased tissue-nonspecific alkaline phosphatase activity and decreased adenosine triphosphate (ATP) production in vascular smooth muscle cells (VSMCs) [44]. As a result, VSMCs' ability to generate extracellular pyrophosphate, a key inhibitor of vascular calcification, is hindered [44]. Villa-Bellosta R et al. used LmnaG609G/G609G to show that inorganic pyrophosphate (PPi) therapy can counteract aortic vascular calcification caused by faulty pyrophosphate synthesis [44].

To investigate the curative prospect of NF-kB suppression on HGPS disease characteristics, Osorio FG et al. discovered that mating Zmpste24-/- mice alongside transgenic mice presenting decreased NF-B signaling improves lifespan and prohibits the progression of progeroid traits [46]. They also demonstrated that sodium salicylate administration effectively suppresses NF-kB activation and related illness symptoms in Zmpste24-deficient mice and improves lifespan in the LmnaG609G/G609G model in the same study [46]. Furthermore, as a result of triggering the reprogramming repressor DOT1L, NF-kB activation inhibits somatic cell reprogramming in aging [47]. The discovery of this molecular process has allowed this information to be translated into a curative method; in progeroid mice, DOT1L inhibition by epz-4777 increased lifespan and avoided aging-related changes [47]. Compound screening, on the other hand, revealed that Remodelin enhances the nuclear shape and fitness in progeric as well as lamin A/C-depleted cells by inhibiting the lamina interacting SUN1-associated acetyl-transferase protein NAT10, as demonstrated by reduction of the DSB markers H2AX and autophosphorylated ataxia telangiectasia mutated (ATM), reduced DNA damage signaling, and enhanced chromatin and nucleolar organization [48]. In HGPS cells, the vitamin D receptor (VDR) levels are reduced and are one of the proteins affected by progerin accumulation [49]. Furthermore, VDR-inactivated mice exhibit a premature aging composition comparable to that seen in HGPS patients [49]. According to Kreienkamp R et al., restoring VDR signaling with 1,25-dihydroxyvitamin D3 (1,25D), the active hormonal form of vitamin D, enhances HGPS symptoms such as DNA repair deficiencies, premature senescence, and nuclear morphological abnormalities [49]. Lamin A/C is another significant progerin target [50]. Progerin does have a substantial binding affinity for lamin A/C, which has a negative dominating effect [50]. Lee SJ et al. used this information to discover a novel chemical (JH4) that can disrupt the connection between progerin and lamin A/C by interacting directly alongside progerin [50]. Nuclear deformation was reduced, and senescence and growth arrest markers were reversed after treatment with JH4 [50]. Furthermore, giving JH4 to LmnaG609G/G609G improved various progeria traits like body weight, cell density, grip strength, and organ size, extending their longevity [50]. A new therapy for progeria has been proposed: resveratrol, a SIRT1 activator that interacts with lamin A [51]. Indeed, because of the obstructing dominating action by progerin, SIRT-1 is delicately linked to the nuclear matrix in the premature aging mouse model Zmpste24-/-, resulting in diminished deacetylase activity and rapid depletion of adult stem cells [51]. Resveratrol eases progeroid characteristics and restores adult stem cell decline in Zmpste24-/- mice by boosting SIRT1 interaction with lamin A [51]. Even though the current model replicates phenotypic progeroid syndromes, the fundamental molecular process is not the same as progeria [52]. To assess the influence of resveratrol on disease phenotypic reversal, more research will be needed employing a model that generates progerin utilizing the same aberrant splicing mechanism seen in humans as the LmnaG609G/G609G model [52]. However, because of its low bioavailability and variable dose-related effects, resveratrol's efficacy has been limited, limiting the full potential of SIRT1 activation therapy [53]. Therefore, SRT501, a new micronized resveratrol formulation with increased bioavailability, tolerance, and pharmacologic efficacy, has been produced [53]. SRT501 has paved a new and exciting research avenue for this family of compounds in developing novel progeria therapeutics [53]. 

Autophagy-activating Drugs

Rapamycin, an immunosuppressive drug used to prevent organ rejection, enhanced the aberrant nuclear shape, lagged the beginning of cellular aging in HGPS fibroblasts, and recovered the chromatin composition of fibroblasts in culture, involving BAF, LAP2alpha distribution patterns, and histone methylation status [54]. Otherwise, research on the LMNA-/- mice muscular dystrophy and dilated cardiomyopathy have shown that rapamycin-mediated therapeutical correction of increased mTORC1 signaling enhances skeletal and cardiac muscle physiology while also increasing lifespan [55]. Furthermore, utilizing the mouse clone, Liao CY et al. discovered that rapamycin administration leads to increased body weight and fat content, as well as longer longevity [56]. Although the findings suggest that LMNA KO mice have a positive effect, this model does not replicate the eugenics and pathogenesis of HGPS in patients [57]. The Clinical and Translational Study Unit (CTSU) of Boston Children's Hospital is now testing everolimus, a rapamycin-derived drug, in conjunction with lonafarnib [57]. Rapamycin suppresses the activity of the mammalian target of rapamycin (mTOR). This protein kinase regulates a wide range of physiological processes such as protein synthesis, cell proliferation, cytoskeleton rearrangements, transcription, immune responses, and autophagy [57]. As a result, extreme caution should be exercised when applying in vitro results to infants with progeria. Additionally, because rapamycin inhibits adipogenesis, caution should be exercised while taking it in HGPS patients with global lipoatrophy and lipodystrophy [57]. In vitro, sulforaphane which is an antioxidant produced from vegetables of the family Brassicaceae, has been shown to improve progerin removal by apoptosis and setbacks the cellular attributes presenting HGPS [58]. The HGPS cellular phenotype was rescued by intermittent treatment with sulforaphane and lonafarnib [59]. Furthermore, because the LMNA promoter consists of a responsive region to retinoic acid, two latest investigations found retinoids in conjunction with rapamycin or alone can lower progerin levels in HGPS patients' skin fibroblasts and restore aging abnormalities [60]. These medications may be helpful in treating progeria, but extensive in vivo research is needed before they can be translated into clinical studies [57].

Prelamin A Isoprenylation and Methylation Inhibitors

During posttranslational processing, the farnesylated carboxy terminus of prelamin A is removed due to the aberrant splicing event that gives rise to progerin [61]. As a result, when progerin dimerizes with wild-type lamins, perpetually farnesylated progerin stays tethered to the inner membrane of the nuclear, causing a cardinal negative breakdown of the nuclear frame [22]. According to this knowledge, blocking farnesylation with farnesyltransferase inhibitor (FTI) medications would reduce progerin synthesis and toxicity [22]. FTIs are tiny compounds that attach to the farnesyltransferase CAAX binding site in a reversible manner [22]. In transitory transfection of HEK 293, HeLa, human HGPS fibroblasts, and NIH 3T3 cells, preventing progerin farnesylation with FTIs regenerated typical nuclear composition as well as led to considerable decreases in nuclear blebbing [22,24]. Furthermore, in recombinant murine HGPS models managed with FTIs, enhanced bone mineralization and weight, increased longevity, and cardiovascular abnormalities are averted [62]. The foregoing investigations led to the start of an anticipated non-randomized clinical trial in 2007, which used an FTI named lonafarnib, which was initially created for cancer treatment [63]. This study included a group of 25 HGPS patients aged 3-16 years old who were given lonafarnib for a minimum of two years [63]. Gordon LB et al. reported that a few children with progeria who were given lonafarnib presented a minor enhancement in weight gain. A 50% increase in weight gain was observed in nine patients, a 50% drop in weight gain in six patients, and 10 patients' rates of weight gain stayed unchanged [63]. Other findings included a reduction of arterial pulse wave velocity by a positional average of 35% in 18 subjects and elevation in skeletal rigidity, bone mineral density, and sensorineural hearing [63]. Even though FTI therapy enhanced an average survival by 1.6 years, it has been described that when farnesyltransferases are inhibited, progerin may become alternatively prenylated by geranylgeranyltransferase I; certainly, the concurrent appearance of an FTI-277, as well as geranylgeranyltransferase I inhibitor (GGTI-2147), resulted in significant levels of prelamin A aggregation when analyzed individually, as a result, the limited benefits of FTI monotherapy can be explained [64]. Therefore, Varela I et al. reasoned that inhibiting both protein farnesylation and geranylgeranylation could reduce the alternate likelihood of prenylation processes conferring FTI defiance [64]. They collaborated with Carlos López-Otín to demonstrate the synergetic reaction of a combination of alendronate (N-BisPhosphonate) and pravastatin (statin) together called ZOPRA on the biosynthetic pathway of farnesyl pyrophosphate, a co-enzyme of farnesyltransferase and a precursor of geranylgeranyl pyrophosphate. Their effectiveness to reduce the prenylation and improvements in growth retardation, weight loss, lipodystrophy, hair loss, and bone abnormalities were observed in the progeroid traits of Zmpste24-/- mice [64]. Similarly, the lifespan of these mice was significantly increased [64]. A phase II, monocentric, open-label, single-arm clinical research employing ZOPRA to investigate the protection as well as adequacy in 12 patients suffering from HGPS for about three and a half years found partly good results, involving bone density abnormalities and weight gain but no serious side effects [65]. Despite the fact that trials utilizing lonafarnib and ZOPRA exhibited some effectiveness for specific criteria, the medications could not be called cures. Hence, a deeper study was required to develop more successful curative access for patients [65]. Nevertheless, awareness is encouraged when using FTIs as a long-term treatment plan, as non-farnesylated prelamin A buildup has been reported to produce fatal cardiomyopathy in mice models of progeria [65].

Downregulation of Prelamin A Aberrant Splicing

Progerin is secluded in atypically formed promyelocytic-nuclear bodies (PML-NB), which Harhouri K et al. discovered as novel progeria biomarkers [66]. They discovered that MG132 inhibits progerin breakdown [66]. In HGPS patient fibroblasts and HGPS patient iPSC-derived mesenchymal stem cells (MSCs) and vascular smooth muscle cells (VSMCs), MG132 causes progerin nucleocytoplasmic translocation following transformation across the nucleolus and progerin removal by macro apoptosis (VSMCs) [66]. In HGPS fibroblasts, MG132 therapy enhances the characteristics of HGPS cells, lowers cell aging, and increases proliferation and potentiality [66]. Furthermore, progerin expression in skeletal muscle of LmnaG609G/G609G mice is reduced in vivo after treatment with MG132 [66]. Overall, they showed that MG132 action reduces progerin and sheds light on a promising family of compounds that could be used as a viable therapy for children with HGPS [66]. The efficacy of an antisense curative access involving morpholino antisense oligonucleotides (AON) in spatially limiting the atypical LMNA splicing section that leads to producing progerin protein has previously been demonstrated ex vivo on cells obtained from HGPS patients and in vivo on a knock-in LmnaG609G/G609G mouse model [67]. Certainly, Osorio FG et al. investigated the synergetic effect of administrating two AONs: "MmEx11," which targets the progeria mutation-activated exon 11 atypical splice section for the sake of preventing its usage, and "MmEx10," which targets the functional exon 10 splice section that aims in strengthening the initial AON's activity by shifting splicing events toward lamin C production [67]. Lee JM et al. got similar results employing a different AON (ASO), which inhibits the binding of the splicing factor SRSF-2 to exon 11 LMNA pre mRNA in the same LmnaG609G/G609G animal model and in HGPS fibroblasts, supporting the efficiency of the method [68].

Humans with various LMNA transmutations that disrupt exon 11 splicings are referred to as "HGPS-like" patients [69]. Progerin and/or additional truncated Prelamin A isoforms (35 and 90) are also produced [69]. Harhouri K et al. recently illustrated that antisense therapeutics could be used to downregulate progerin and other shortened or mutated Prelamin A isoforms in HGPS-like and mandibuloacral dysplasia type B (MAD-B) patient cells. It shows a piece of preclinical evidence essential for the usage of antisense morpholino oligonucleotides in MAD-B, HGPS, and HGPS-like syndrome [69]. Nevertheless, because of their known toxicity, administering identical compounds to mice which as Vivo-morpholinos, could not be considered for children [69]. For future therapeutic approaches utilizing splicing modulation, the choice of AON chemistry and route of delivery will be critical [69]. The HGPS mutation causes an internal 5' cryptic splice site within exon 11 of the LMNA pre-mRNA to be used, resulting in atypical alternative splicing and generation of a shortened version of prelamin A in human HGPS primary fibroblasts and mice LmnaG609G/G609G 56 (progerin) [70]. Serine/Arginine-rich splicing factor 1 (for SRSF-1), an RNA-binding protein, was discovered to promote this abnormal alternative splicing in 2011 [70]. On the other hand, the anti-diabetic medicine Metformin has been demonstrated to influence SRSF-1 expression through transcription [71]. Based on these findings, Metformin reduces SRSF-1 and progerin expression in MSCs derived from HGPS-involved hematopoietic stem cells (HGPS MSCs) as well as in various ex vivo HGPS models like human primary HGPS fibroblasts and LmnaG609G/G609G mouse fibroblasts, according to Egesipe AL et al., succeeded in enhancing nuclear morphology as well as untimely differentiation of osteoblasts of HGPS MSCs [71].

The introduction of CRISPR therapies has sparked hopes for a genetic approach by reducing progerin accumulation [72]. A Cas9 endonuclease is guided by a single-guide RNA (sgRNA) that recognizes its target region as well as a protospacer adjacent motif (PAM) in the CRISPR/Cas9 system [72]. The nuclease causes double-strand breaks in DNA, often repaired by non-homologous end-joining, resulting in insertions and deletions (indels) [72]. Santiago-Fernández O et al. conducted a study aimed at blocking the accumulation of progerin by a CRISPR/Cas9-based strategy against HGPS [72]. The LMNA gene encodes lamin C (exons 1-10) and lamin A (exons 11-12) via alternative splicing and polyadenylation [72]. Because lamin A/progerin development appears to be dispensable, the aim is to disrupt the final section of the LMNA gene, limiting lamin A/progerin development without harming lamin C [73]. The findings indicate that a one-time IV treatment with CRISPR/Cas9-mediated lamin A/progerin reduction improves the health and lifespan of HGPS mice [73]. As a proof of concept, transgenic mice expressed Streptococcus pyogenes Cas9 and delivered the gRNAs exogenously [73]. As a result, there is a potential for CRISPR/Cas9 to be used in the care of human HGPS patients [73]. Even though this technique disrupts both lamin A and progerin expression, knockout experiments in mice show that mice lacking lamin A are viable and even live longer than wild-type mice [73]. Given that HGPS is a fatal disease, this approach to extending patients' survival and longevity is an appealing mode of action [73]. Surprisingly, even though a minor therapeutic effect was observed, this result was associated with gene editing occurring primarily in the liver and, to a lesser extent, in other organs [73]. More broad-spectrum targeting, such as targeting the colon, or combination therapy with FTIs, may help HGPS patients live longer [73].

## Conclusions

HGPS is a segmental "premature aging" condition in which children show phenotypes that may reveal information about the aging process at both the cellular and organismal levels. Over the years, substantial progress has been made in understanding the disease, yet much more is to be learned. As the study progresses, HGPS continues to uncover previously unknown aspects of aging. With all of its complexities, HGPS provides a unique model for clarifying new lamin A/C and progerin roles in the cell. Progeria research has resulted in an increasing number of intriguing treatment possibilities. It is worth noting that in HGPS preclinical investigations, a drug's potential to selectively choose the cardiovascular system should be assessed, as it's the disorder's main functional aim, resulting in premature death. Cardiovascular measurements would also be selected among treatment efficacy readouts. In order to uncover new therapy pathophysiology targets and other outcome metrics, it will be necessary to improve our understanding of disease biology. This will allow us to understand better which damaged pathways are most relevant to the disease. We recommend that more future studies be performed to uncover the treatment efficacies of current and upcoming management modalities.
